# Learning to live with RA as an unwelcome companion—patients’ experiences after 1 to 2 years of DMARD treatment

**DOI:** 10.1016/j.ero.2026.100238

**Published:** 2026-09-10

**Authors:** Ingrid Larsson, Ellen Landgren, Elisabet Lindqvist, Elisabeth Mogard, Maria Nylander, Ann Bremander

**Affiliations:** 1 Department of Health and Care, School of Health and Welfare, Halmstad University, Halmstad, Sweden; 2 Spenshult Research and Development Centre, Halmstad, Sweden; 3 Department of Clinical Sciences, Section of Rheumatology, Lund University, Lund, Sweden; 4 Department of Rheumatology, Skåne University Hospital, Lund, Sweden; 5 Swedish Rheumatism Association, Stockholm, Sweden

## Abstract

**Objectives:**

Rheumatoid arthritis (RA) can be life-changing, requiring long-term treatment to control disease activity. It is therefore important to gain knowledge about patients’ experiences of living with RA after 1 to 2 years of disease-modifying antirheumatic drug (DMARD) treatment.

**Methods:**

This qualitative study used an inductive approach. Interviews were conducted with 22 patients with RA in 7 focus groups (17 participants) and 5 individual interviews. Participants were 15 women and 7 men, with a median age of 57 years, an RA disease duration of 12 to 21 months, and 12 to 20 months of treatment with conventional synthetic or biological DMARDs. The interviews were analysed using qualitative content analysis.

**Results:**

Patients’ experiences of living with RA after 1 to 2 years of DMARD treatment reflected a recognition of RA as an unwelcome companion that the patients had learned to live with. The experiences were expressed as: (i) managing daily life despite disease limitations, including struggling with work-life balance and trying to maintain social participation; (ii) reconfiguring the self with disease limitations through feeling unlike oneself, and experiencing changes in bodily appearance and mood; (iii) finding humility despite disease limitations by being grateful for the treatment and making the most of each day.

**Conclusions:**

After 1 to 2 years of DMARD treatment, participants experienced RA as an unwelcome companion, with daily life constrained by physical, psychological, and social demands. Care should therefore extend beyond inflammation control to include person-centred, goal-oriented support and rehabilitation.

**WHAT IS ALREADY KNOWN ON THIS TOPIC**

• Despite improved outcomes with disease-modifying antirheumatic drugs (DMARDs) and treat-to-target strategies, many people with rheumatoid arthritis (RA) continue to experience fatigue and difficulties in everyday life.
• Little is known about how patients experience living with RA after 1 to 2 years of DMARD treatment, when treatment is established but adaptation to everyday life is still ongoing.

**WHAT THIS STUDY ADDS**

• After 1 to 2 years of DMARD treatment, patients described RA as an unwelcome companion that they had learned to live with rather than something fully resolved by treatment.
• Patients continued to struggle with work-life balance, social participation, sense of self, mood, and changes in bodily appearance, while also expressing gratitude for treatment and trying to make the most of each day.

**HOW THIS STUDY MIGHT AFFECT RESEARCH, PRACTICE OR POLICY**

• Follow-up in RA should extend beyond disease activity to include regular dialogue about everyday functioning, work, social participation, bodily appearance, mood, and sense of self.
• Future research and care should give greater attention to person-centred, nonpharmacological and rehabilitation support that helps people maintain functioning, participation and everyday life.

Alt-text: Unlabelled box dummy alt text

## BACKGROUND

Living with rheumatoid arthritis (RA) has an impact on physical, psychological, and social well‑being across the life course [[1], [2], [3]]. Disease-modifying antirheumatic drugs (DMARDs), used with a treat-to-target approach involving regular monitoring and treatment adjustments to achieve low disease activity or remission, have improved patient outcomes [4]. The goal of treatment is to achieve optimal health and as normal a life as possible [4]. Still, RA can be life-changing because disease control often requires long-term treatment. Despite optimal treatment, about two-thirds of patients report clinically significant, and often persistent, fatigue [5]. Nonpharmacological interventions, such as exercise and cognitive behavioural therapy, can provide small to moderate improvements in fatigue and pain [5,6]. Adapting to life with a chronic disease is a multidimensional, challenging process [7], and stronger social support has been associated with better treatment adherence [8] and quality of life [9]. In the early disease phase, patients focus on ‘mastering a new life situation’ and seek help managing fluctuating disease symptoms, whereas in later phases, support that facilitates self-management is increasingly desired [10,11]. Adaptation to chronic disease has also been linked to resilience. People living with RA often use a combination of emotional and behavioural self-management strategies [12]. By recognising patients’ self-management needs, healthcare professionals might be able to better support self-management and empowerment [13,14].

Patients who have lived with RA for many years have described several important aspects affecting daily life, such as symptoms and functional limitations, increased risk of comorbidities, effects on close relationships and family roles, including intimacy and caring responsibilities, participation restrictions, and the impact of illness perceptions, social support, and financial burden [2,3,15,16]. Different stages of living with RA may involve different experiences and strategies. It is therefore important to gain knowledge about how patients experience everyday life during the first years of DMARD treatment. Hence, the aim of this study was to describe patients’ experiences of living with RA after 1 to 2 years of DMARD treatment.

## METHODS

### Study design

This study had a qualitative design and used inductive qualitative content analysis [17,18]. Reporting followed the Consolidated Criteria for Reporting Qualitative Research 32-item checklist [19].

### Participants and recruitment

This study is based on the Swedish data from the broader European Qualitative research project on Patient-preferred outcomes in Early RA (EQPERA) study, described elsewhere [20]. Patients were recruited from 2 rheumatology clinics in 4 cities in southern Sweden, representing both university hospitals and regional rheumatology specialist outpatient clinics, with catchment areas covering both urban and rural areas. Treatment followed national and international guidelines for RA management, with DMARD treatment provided as part of routine rheumatology care [4]. For the present analysis, eligible participants were adults with RA fulfilling the 2010 American College of Rheumatology/European League Against Rheumatism (EULAR) classification criteria [21], who had lived with an RA diagnosis for 1 to 2 years and had participated in an earlier EQPERA interview 6 to 12 months before the present interview round. All 31 eligible patients were invited; 22 accepted and 9 declined further participation for personal reasons. The final sample comprised 15 women and 7 men, with a median age of 57 years and 12 to 20 months of DMARD treatment. Participant demographic, clinical, and self-reported health characteristics are presented in Table 1.

**Table 1:** Participant demographic, clinical, and self-reported health characteristics

No. of participants (n)	22
Individual interviews	5
Focus group interviews	17
Site of recruitment	
University hospital	20
Regional rheumatology specialist outpatient clinic	2
Sex	
Female/Male (n)	15/7
Age (y) Median (range)	57 (42–81)
Civil status (n)	
Cohabiting/living alone	19/3
Education Level (n)	
Primary School/Secondary/University	5/11/6
Employment (n)	
Employed/Student/Unemployed/Retired	12/0/0/10
Disease duration (mo) Median (range)	18 (12-21)
DAS28CRP score^a^ Median (range)	2.1 (1.2-3.4)
Current RA treatment (n)	
csDMARDs	16
bDMARDs	6
Discontinued treatment	3
DMARD treatment duration (mo) Median (range)	17 (12-20)
General health, VAS (0-100 mm)^b^ Median (range)	25 (0-95)
Pain, VAS (0-100 mm)^b^ Median (range)	20 (0-50)
Fatigue, VAS (0-100 mm)^b^ Median (range)	40 (0-100)

bDMARD, biological disease-modifying antirheumatic drug; csDMARD, conventional synthetic disease-modifying antirheumatic drug

a DAS28CRP, Disease Activity Score in 28 joints using C-reactive protein; available for 18/22 participants. Closest assessment to the interview: median 2.5 months (range 0-9). For the 4 missing values, 2 participants were noted as being in remission in the medical record and 2 were lost to follow-up.

b During the past week. VAS, visual analogue scale, 0 to 100 mm; higher scores indicate worse general health, pain, and fatigue.

### Data collection

Data were collected in 2018 through 7 face-to-face focus groups, each including 2 to 3 participants, and 5 individual interviews. Focus groups were prioritised to capture interaction and shared meaning-making, whereas individual interviews were used when participants were unable or preferred not to join a group. Of the 5 individual interviews, 3 were conducted face-to-face at the rheumatology clinic and 2 were conducted by telephone due to geographical distance. The interviews were conducted by 2 female rheumatology nurses (IL and ELa), both with clinical experience in rheumatology care and training in qualitative interviewing. IL is an experienced qualitative researcher in rheumatology and supervised ELa throughout data collection to support methodological consistency. Neither interviewer had a prior relationship with the participants. The same semistructured interview guide with open-ended questions was used across both formats. Focus groups took place in quiet, separate rooms where conversations could not be overheard, either at the rheumatology clinics or at a regional research and development centre. Focus groups lasted 54 to 110 minutes (median 78), and individual interviews lasted 20 to 58 minutes (median 35). All interviews were audio-recorded, transcribed verbatim, conducted in Swedish, and anonymised before analysis.

### Data analysis

A qualitative content analysis was conducted with iterative movement between the whole text and its parts [17,18]. All transcripts were read repeatedly to achieve familiarity. Text segments relevant to the aim were identified as meaning units across both focus-group and individual interviews. In total, 469 meaning units were condensed while preserving core content, coded, and compared to identify similarities, differences, and contrasting accounts across the dataset and between interview formats. The codes were grouped into subcategories, which were abstracted into categories representing the manifest content. Finally, an overarching theme was formulated to express the latent meaning across the categories (Table 2). Emerging interpretations were continuously checked against the full transcripts, and earlier analytical decisions were revisited when required, to ensure coherence and conceptual fit.

**Table 2:** Overview of the theme, categories, subcategories, and quotations illustrating patients’ experiences of living with RA after 1 to 2 years of DMARD treatment

Theme	Categories	Subcategories	Quotations
RA as an Unwelcome Companion: Learning to Live Together	Managing daily life despite disease limitations	Struggling with work-life balance	‘On Saturdays, I usually sleep since I’m working all week. So Sunday is the only day I have energy for cleaning’ [FG 1]
			‘I am on the roads driving trucks. I need to be very much awake. So I need to plan the morning, do I have time to rest now…all the time planning to have enough energy’. [FG 3]
		Trying to maintain social participation	‘Life is not fun for the person you live with…My husband also felt very lonely and it was not fun for him either. Because I was not the person I was before’. [FG 2]
			‘…I get so much support … that’s why I feel extra good today’. [I 5]
	Reconfiguring the self with disease limitations	Feeling unlike oneself	‘I was always the one who was out and about before, but I can’t manage that anymore’. [FG 1]
			‘I felt trapped in my own body. I was not free; I did not feel the freedom to get up and do something’. [FG 2]
		Experiencing changes in bodily appearance	‘Right now, the most important thing for me is not to lose my hair… to be completely honest, I am that vain’. [I 4]
			‘With the weight, I hate that I only go upward’. [FG 6]
		Experiencing mood changes	‘It is depressing, simply… I would rather have pain than be tired’. [FG 7]
			‘On Thursdays … very, very irritable, I can hardly manage anything. At home, they now know that when Dad has taken his medicine, there is no point in discussing things with him’. [I 1]
	Finding humility despite disease limitations	Being grateful for the treatment	‘Now that I receive Enbrel … without it I would not have been able to live, the pain was so severe’. [I 3]
			‘It is as if I have got my life back again. I can do whatever I want’. [I 4]
		Making the most of each day	‘Yesterday was yesterday and tomorrow is tomorrow … make sure to live today’. [FG 2]
			‘We go out and walk in the morning… about 5 km… and it feels good’. [FG 5]

DMARD, disease-modifying antirheumatic drug; FG, Focus group; I, individual interview; RA, rheumatoid arthritis.

Primary coding and the initial analytical structure were developed by the first and last authors (IL and AB), after which the coding framework, subcategories, categories, and theme were discussed within the multidisciplinary research team until consensus was reached. The team included researchers with clinical and methodological expertise in rheumatology nursing and qualitative research (IL and ELa), physiotherapy and rehabilitation (AB and EM), and medicine/rheumatology (ELi), together with a patient research partner (MN) who participated as a full member of the research team in the analytical discussions and decisions.

### Ethical considerations

The study was approved by the Regional Ethical Review Board in Lund, Sweden (nos. 2016/618, 2017/205), and conducted in accordance with the ethical principles of the Declaration of Helsinki [22]. All participants provided written informed consent. Two of the interviewing authors (ELa and IL) provided the participants with oral and written information about the study, voluntary participation, and the right to withdraw at any time without giving a reason or affecting their healthcare or treatment.

### Patient and public involvement

This study was conducted in accordance with the EULAR recommendations for involving patient research partners in rheumatology research [23]. One patient research partner (MN) with lived experience of rheumatic and musculoskeletal disease participated throughout the research process. MN provided feedback on the study design and interview guide with particular attention to whether the questions were understandable and relevant from a patient perspective. MN also participated in discussions of the emerging categories and theme and contributed to analytical decisions about the wording and interpretation of the findings. This input helped strengthen the relevance of the findings and supported the presentation of the results and conclusions from a patient perspective.

## RESULTS

Patients’ experiences of living with RA after 1 to 2 years of DMARD treatment involved recognising RA as an unwelcome companion that they had learned to live with. Across the categories, this recognition was reflected in how RA shaped daily routines, sense of self, and ways of approaching treatment and everyday life. The experiences were expressed as: (i) managing daily life despite disease limitations, (ii) reconfiguring the self with disease limitations, and (iii) finding humility despite disease limitations. The overarching theme, categories, and subcategories are presented in Table 2 and illustrated by quotations from the interviews.

### Managing daily life despite disease limitations

Managing daily life despite disease limitations was expressed as struggling with work‑life balance and trying to maintain social participation. Patients described ongoing adaptations in priorities, boundaries, and expectations to maintain participation in work and relationships as energy levels fluctuated.

#### Struggling with work-life balance

Patients struggled to find a new work-life balance in managing daily life. Routines were changed, and more time was needed for ordinary tasks, both at work and at home. Fatigue affected both work and recovery, as 1 participant described: ‘Work is demanding… but my real problem is that I am completely exhausted when I get home… I’ve become unsociable’ (Focus group 7). Pain, impaired sleep quality, morning stiffness, impaired range of motion, and reduced muscular strength also restricted everyday activities and sometimes forced patients to surrender to the limitations set by the disease.

Medication could also affect work-life balance, for example, through adverse effects such as nausea associated with weekly methotrexate. Work was often prioritised over exercise or rest, which further complicated the balance between work, recovery, and everyday activities.

#### Trying to maintain social participation

Living with RA affected patients’ social participation and ability to maintain independence, making daily life management challenging. Unpredictable fatigue restricted social life and required careful planning, as 1 participant described: ‘So there is less socialising, you have less energy’ (Individual interview 2). Interactions with family and friends were affected, and some patients described withdrawal, procrastination, or isolation as ways of adapting to limited energy. At the same time, social life was adapted rather than abandoned. Patients described low-threshold activities, such as exercising with others, pacing invitations, meeting in smaller or closer circles, and drawing on family support as ways to maintain connection while acknowledging limits.

### Reconfiguring the self with disease limitations

Reconfiguring the self with disease limitations was reflected in feeling unlike oneself, experiencing changes in bodily appearance, and experiencing mood changes, all of which were shaped by living with RA as an unwelcome companion. Patients described shifts in how the self was seen in relation to symptoms and treatment, as capacities changed, appearances altered, and feelings fluctuated, whereas efforts continued to maintain a sense of continuity in everyday life.

#### Feeling unlike oneself

Feeling unlike oneself captured the sense of mismatch between a previously familiar self and current capacities. Changes in energy, clarity, and sense of meaning meant that plans did not always translate into action, routines took longer to initiate, and formerly automatic roles required pacing or assistance. The condition could be largely invisible to others, yet deeply felt from within: “Even though I do not look sick, I am. But they do not think so… ‘you look healthy and fresh today’ – You should have seen me on the inside” (Focus group 3). Patients described feeling unlike their former selves when fatigue, pain, shakiness, or cognitive strain altered their initiative and social engagement. This did not signal resignation, but rather an ongoing adaptation of identity in which patients tried to regain a coherent sense of ‘who I am’ in daily activities and relationships.

#### Experiencing changes in bodily appearance

Experiencing changes in bodily appearance described how visible and invisible bodily changes intersected with the reconfiguring of the self, as patients learned to live with RA as an unwelcome companion. Patients described hair loss, memory loss, dizziness, nausea, shakiness, fear of falling, weight change, facial puffiness, swelling in hands or feet, easy bruising, and dry eyes or mouth as everyday signs that their bodies no longer looked or felt as they had before—bodily appearances that challenged their sense of self. Patients expressed the need to adapt their clothing to hide bruises or swelling, to adjust hairstyles or cover thinning hair, and at times to reconsider medication when appearance felt central to self‑presentation and self‑identity. Some also weighed tradeoffs; for instance, accepting more pain if hair loss could be avoided, whereas others tried to maintain participation through practical adjustments and acceptance.

#### Experiencing mood changes

Experiencing mood changes, including low mood, depression, melancholy, and mood swings, was linked to disease-related limitations and loss of abilities, which affected patients’ sense of self. Patients described sadness, loss of motivation, and difficulty initiating everyday activities, often in connection with fatigue, sleep disturbance, and pain. In this reading of reconfiguring the self, mood changes undermined patients’ usual motivation and ability to initiate everyday activities. Day‑to‑day continuity and plans sometimes gave way to withdrawal or rest. Patients expressed that feeling down could make even ordinary interactions or plans feel heavy, shaping how the self was seen in everyday life, requiring pacing and support to maintain engagement. Moods shifted with changes in roles and social participation. Patients continued to work towards regaining a coherent sense of ‘who I am’, despite periods of low mood.

### Finding humility despite disease limitations

Finding humility despite disease limitations was reflected in being grateful for the treatment and making the most of each day, reflecting the experience of living with RA as an unwelcome companion. Patients acknowledged persistent constraints and described how treatment and everyday choices helped maintain function and, when possible, regain valued roles and activities.

#### Being grateful for the treatment

Patients expressed gratitude for the treatment while at the same time describing ongoing disease-related limitations and an awareness of the increased risk of RA comorbidities. They described accepting the chronic nature of the disease and realising that ‘it could have been worse’. Treatment was valued for relieving pain, swelling, and stiffness and for supporting mobility and everyday activities. As 1 participant expressed it: ‘It just gets better and better; it is so nice to have medicines that work’ (Focus group 4). Patients also valued structured follow-up, timely contact with clinical staff during flares, and access to allied healthcare professionals. A stable, workable day-to-day life was regarded as meaningful, even when some symptoms or side effects remained.

#### Making the most of each day

Patients expressed concerns about the future and no longer took good health for granted. At the same time, they tried, as best they could, to make the most of each day. This involved setting simple routines, planning their energy use, and keeping up regular exercise, even when symptoms were present. One participant described this by saying: ‘I have started to exercise seriously. I go to the gym 3 times a week… regardless of how tired I am, I go to the gym and do a little’ (Individual interview 1). Making the most of each day also meant adapting habits, modifying tasks at home or at work, and planning around weather or symptom patterns to remain engaged in valued activities.

## DISCUSSION

The findings showed that living with RA after 1 to 2 years of DMARD treatment involved recognising RA as an unwelcome companion that had to be incorporated into everyday life. This was reflected in 3 interconnected dimensions: managing daily life despite disease limitations, reconfiguring the self with disease limitations, and finding humility despite disease limitations. Together, these findings indicate that treatment benefits may coexist with residual symptoms, treatment-related side effects, fatigue, and fluctuating capacity, with ongoing impact on everyday life. The study therefore highlights a phase in which pharmacological treatment has been established, but adaptation to everyday life remains ongoing.

Adaptation to living with RA posed challenges in everyday life, as patients struggled to balance work and social participation despite disease limitations. Although work loss in RA has decreased with improved pharmacological treatment, work-loss days remain more common than in the general population [24]. Contemporary registry data further suggest that work-loss trajectories are broadly similar across biologic DMARD classes, with levels peaking around treatment initiation and then stabilising over time [25]. Reduced work ability is common in RA and is associated with lower quality of life [26]. At the same time, many people with RA strive to remain in work despite fatigue and limited energy and report unmet needs for tailored vocational support [27]. EULAR has emphasised that work participation should be addressed in healthcare consultations and supported through attention to work-related limitations and flexible workplace conditions [28], and that it may be beneficial in terms of health outcomes [29]. Supporting work participation may therefore be important not only for continued employment but also to achieve a better balance in everyday life, particularly when support is introduced early and tailored to individual needs [30].

RA also restricted social participation by reducing patients’ energy available to engage with family and friends and to take part in valued leisure activities. Patients with RA continue to experience a range of challenges to social participation related to living with a chronic rheumatic disease [31], throughout the disease course [32]. Research further suggests that illness perceptions may shape social participation in RA, indicating that participation is influenced not only by symptoms but also by how the disease is understood and managed in everyday life [33]. In the present study, however, social participation was not only reduced but also adapted. Patients described limiting invitations, choosing low-threshold activities, and relying on family support to remain socially connected despite fluctuating energy. This aligns with research showing that social participation in RA is associated with satisfaction with social roles, greater mobility, lower social isolation, instrumental support, and lower disease activity [34]. The findings also indicated that support from others was important, as support from family and social networks made continued participation more manageable. This aligns with evidence suggesting that positive social support, particularly from family and friends, is important for quality of life and well-being among people with RA [9].

The patients in the present study experienced a reconfiguration of the self. It was described as a mismatch between a previously familiar self and current capacities, as well as changes in bodily appearance and mood. A systematic review concluded that people with RA had to ‘renegotiate the self’ to facilitate self-management, and that self-concept, self-esteem, and self-efficacy were cognitive-emotional experiences affected by the disease [35].

The bodily changes described by patients may help explain why self-identity was impacted after pharmacological treatment had been established. RA may influence physical functioning over time [36], involve extra-articular manifestations such as ocular symptoms, most commonly dry eye or keratoconjunctivitis sicca [37], and give rise to treatment-related side effects including nausea, hair loss, and weight gain [38]. Together, these changes may shape how the body is experienced and managed in everyday life, thereby contributing to changes in self-identity [39,40].

The findings also reflected the challenge of living with a disease that was at times invisible to others while profoundly affecting how the self was experienced from within. Previous qualitative research has shown that RA may alter patients’ sense of identity both in private and in public, particularly when bodily changes, fatigue, and fluctuating abilities create a mismatch between the former and current self [41,42]. Treatment-related side effects and bodily changes may further complicate this change, as patients have reported a need for support in coping with changes in their bodies and psychological well-being [16]. Low mood may also be part of the adaptation process, as emotional distress and reduced psychological well-being can shape how life with RA is experienced and managed in everyday life [16,35].

Despite persistent disease limitations, the findings highlighted patients’ gratitude for effective treatment and a willingness to make the most of everyday life. Being grateful for treatment was linked to experiences of symptom relief, regained mobility, and the possibility of maintaining participation in everyday life and valued activities. This aligns with research showing that, even with contemporary treatment, everyday life with RA still involves ongoing adaptation to maintain participation in valued activities [43]. Acceptance may help to understand this pattern, as acceptance in RA has been described as a gradual and nonlinear process of integrating the disease into daily life, rather than simply overcoming it [44]. Acceptance of living with the disease has also been described as an important part of adaptation in RA [45].

Making the most of each day appeared to reflect this same process of adaptation. Patients described pacing, planning, regular exercise, and small, manageable steps as ways of preserving function and staying engaged in everyday life. This corresponds with previous research showing that maintaining physical activity in RA often requires adaptation to fluctuating symptoms, individual circumstances, and changing conditions in everyday life [46]. Lifestyle discussions in rheumatology care have also been described by people with RA as supporting self-management in everyday life, which aligns with how patients in the present study linked everyday choices to maintaining function and valued roles [47]. Taken together, the findings suggest that finding humility despite disease limitations is better understood as a pragmatic form of adaptation, rather than as resignation [44].

### Methodological considerations

The trustworthiness of the study was assessed in relation to credibility, dependability, confirmability, and transferability [17,18]. Credibility was strengthened by the inductive qualitative content analysis, with iterative movement between the whole and the parts and a clear progression from meaning units to subcategories, categories, and an overarching theme. The material provided rich and varied descriptions, with recurring patterns across both focus-group and individual-interview data. Variation in participants’ experiences was considered when refining the categories and theme. The combination of small focus groups and individual interviews contributed to variation in the data: focus groups enabled interaction and shared meaning-making, whereas individual interviews created space for more personal accounts. No clear differences in the content of experiences were identified between focus-group and individual-interview data, although the different interview formats may have influenced how experiences were expressed. Dependability was supported by using the same semistructured interview guide across both formats, trained interviewers with no prior relationship to the participants, verbatim transcription, and a stepwise analytical process discussed within the research team. Confirmability was strengthened by the transparent description of the analytic process, the grounding of interpretations in quotations, and discussions within a multidisciplinary research team that included a patient research partner as a full member. The patient research partner contributed to discussions about the relevance and resonance of the categories and theme, which helped ensure that the findings remained meaningful and relevant from a patient perspective. Transferability was facilitated by descriptions of the study setting, participant characteristics, and treatment phase, allowing readers to judge the relevance of the findings in other contexts. Only the later EQPERA interviews were analysed, limiting insight into within-person changes over time. Although Disease Activity Score in 28 joints using C-reactive protein data were available for most participants, these assessments were not systematically collected at the time of the interviews (Table 1). The findings should therefore not be interpreted as representing a uniform disease activity state or as showing direct relationships between disease activity and everyday experiences, but rather as patients’ experiences of everyday life after 1 to 2 years of DMARD treatment with varying self-reported symptom burden. The interviews were conducted in 2018, and developments in RA treatment and care may have influenced patients’ experiences in contemporary Swedish rheumatology care. However, the findings remain relevant because they highlight how treatment benefits may coexist with residual symptoms, fatigue, participation restrictions, and ongoing adaptation to everyday life in a phase in which pharmacological treatment has been established.

### Clinical implications

The findings suggest that established DMARD treatment does not necessarily mean that life has returned to normal for people living with RA. Clinical follow-up, therefore, needs to extend beyond disease activity and include person-centred dialogue about how RA affects work-life balance, social participation, mood, sense of self, and adaptation to life with a long-term disease. Fatigue, reduced energy, and fluctuating ability should be addressed as clinically relevant issues, including when inflammation appears controlled. Person-centred care may help identify valued roles and activities and support realistic strategies for participation, including discussions about work demands and accommodations, support for physical activity and other health behaviours adapted to fluctuating symptoms, and attention to psychological well-being, treatment-related changes in bodily appearance, and rehabilitation needs. In practice, rheumatologists and rheumatology nurses are well placed to identify ongoing concerns and coordinate support from relevant professionals, such as physiotherapists, occupational therapists, psychologists, counsellors, and vocational rehabilitation professionals, when needed. A more holistic follow-up may help bridge the gap between clinical improvement and everyday life.

## CONCLUSIONS

After 1 to 2 years of DMARD treatment, participants continued to experience RA as an unwelcome companion, with daily life constrained by physical, psychological, and social demands. They described an ongoing process of adapting to life with RA while striving to sustain work-life balance, preserve social participation, and reconfigure their sense of self, alongside gratitude for treatment benefits. These findings indicate that, among patients established on DMARD treatment, perceived treatment benefits may coexist with residual symptoms and everyday impact, including reduced functioning, participation restrictions, and changes in self-perception. Care should therefore extend beyond inflammation control to include person-centred, goal-oriented support and rehabilitation, routine dialogue about work demands and accommodations, and support for fatigue, mood, and adaptation in everyday life. Future research should investigate how nonpharmacological and rehabilitation interventions can support functioning, participation, and everyday life, and evaluate outcomes that matter to patients, including daily life, work sustainability, social participation, and self-efficacy, alongside standard clinical outcomes.

## Competing interests

All authors declare they have no competing interests.
